# A practical approach to imaging characteristics and standardized reporting of COVID-19: a radiologic review

**DOI:** 10.1186/s40779-021-00301-y

**Published:** 2021-01-24

**Authors:** Deniz Esin Tekcan Sanli, Duzgun Yildirim, Ahmet Necati Sanli, Suha Turkmen, Neval Erozan, Guray Husmen, Aytug Altundag, Filiz Tuzuner

**Affiliations:** 1 Department of Radiology, Acibadem Kozyatagi Hospital, 19 Mayıs Mah, İnönü Cad, Okur Sok, No.24/A, Kozyatağı, 34734 Istanbul, Turkey; 2 Department of Radiology, Acibadem Taksim Hospital, 34373 Istanbul, Turkey; 3 Cerrahpasa Medical Faculty, Department of General Surgery, Istanbul University-Cerrahpasa, 34098 Istanbul, Turkey; 4 Department of Emergency, Acibadem Taksim Hospital, 34373 Istanbul, Turkey; 5 Department of Otolaryngology, Acibadem Taksim Hospital, 34373 Istanbul, Turkey; 6 Department of Anesthesiology, Acibadem Taksim Hospital, 34373 Istanbul, Turkey

**Keywords:** COVID-19, SARS-CoV-2, Computed tomography, Pneumonia, Acute respiratory distress syndrome, Ground-glass opacities, Crazy-paving pattern

## Abstract

Novel coronavirus (2019-nCoV), also known as severe acute respiratory syndrome coronavirus 2 (SARS-CoV-2), is a pathogen that has caused a rapidly spreading pandemic all over the world. The primary mean of transmission is inhalation with a predilection for respiratory system involvement, especially in the distal airways. The disease that arises from this novel coronavirus is named coronavirus disease 2019 (COVID-19). COVID-19 may have a rapid and devastating course in some cases leading to severe complications and death. Radiological imaging methods have an invaluable role in diagnosis, follow-up, and treatment. In this review, radiological imaging findings of COVID-19 have been systematically reviewed based on the published literature so far. Radiologic reporting templates are also emphasized from a different point of view, considering specific distinctive patterns of involvement.

**Supplementary Information:**

The online version contains supplementary material available at 10.1186/s40779-021-00301-y.

## Background

As of December 28, 2020, the novel coronavirus infected more than 80.45 million people worldwide and caused more than 1.77 million deaths [1]. The disease progresses asymptomatically or mildly in the majority, with the most common form of presentation as fever and upper respiratory infection symptoms [1, 2]. However, in the presence of comorbid diseases or advanced age, the disease may aggravate and lead to complications, such as pneumonia, acute respiratory distress syndrome, multiorgan failure, and death, depending on the degree of diffuse alveolar damage and inflammatory response [3]. Radiology plays a significant role in the diagnosis, follow-up, and treatment, with radiological imaging becoming increasingly more important in patient management [4].

## Diagnosis of the disease

The diagnosis of COVID-19 is made by evaluating clinical examination and laboratory findings together with contact history and time [5]. Direct radiography (X-ray) and chest computed tomography (CT) are used to support the diagnosis inappropriate indications. As indicated by national and international radiology associations, methods using X-ray should not be used for scanning purposes [6].

### X-ray radiography

**Fig. 1:**
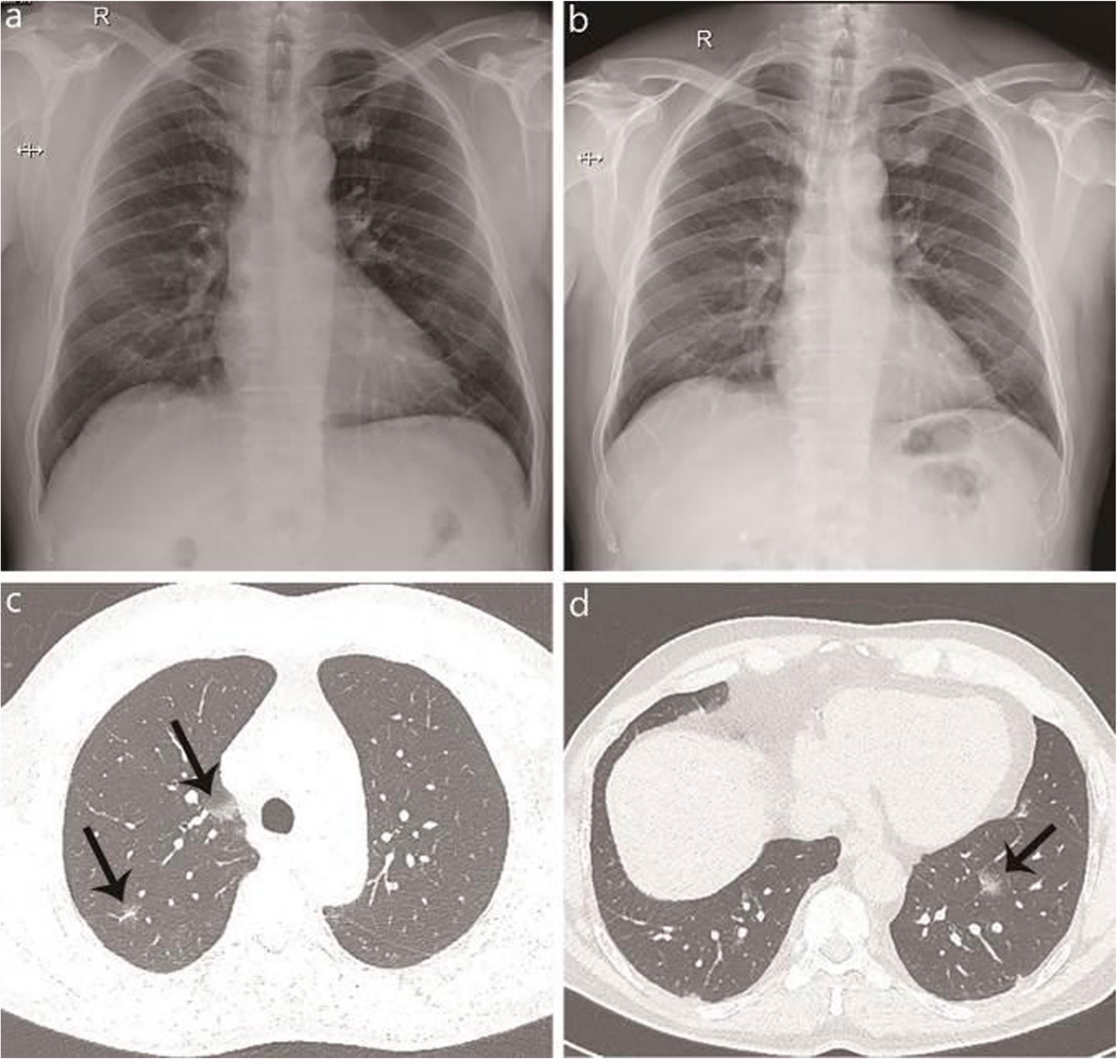
The importance of CT in showing the ground-glass opacities common in disease. **a**. PA chest radiograph of a 66-year-old male patient in 2016. **b**. chest radiograph on March 23, 2020. There is no significant difference between the two graphics. The chest radiography is mostly normal, especially during the early disease period. **c-d**. CT of the same patient on the same date as the second X-ray (March 23, 2020) shows ground-glass opacities (black arrows) in the right middle and bilateral lower lungs and findings were suspicious for COVID-19. The sensitivity of chest radiograph is always lower in demonstrating ground-glass opacities of these sizes and low density

Due to the nature of the disease, COVID-19 often begins as ground-glass opacity as an imaging finding reflecting underlying alveolar inflammation [7]. The diagnostic sensitivity of the chest radiography is relatively low to show these ground-glass opacities (25–60%) in the studies conducted on cases with clinically significant findings (Fig. 1) [3, 8–10]. The routine use of CT scanning for follow-up purposes in daily practice is hampered by the relatively high radiation dose. Therefore, COVID-19 is used serially in the radiological follow-up of patients with initial positive CT findings, especially to assess for developing consolidations in patients with poor prognostic factors [11].

### Ultrasonography

Although the effectiveness of ultrasound is controversial, it can be used in selected cases for its ability to detect effusion and wide consolidations. Additionally, ultrasound allows effusion drainage in the same session in immobile progressive patients, especially in intensive care units [12].

### Ct

Although CT is more likely to be positive after the onset of symptoms (especially after 3 days of symptoms onset), CT findings can even be seen during the asymptomatic period [13]. CT positivity 3 days before real time-polymerase chain reaction (RT-PCR) positivity has been reported in some studies [14, 15].

## CT findings

**Fig. 2:**
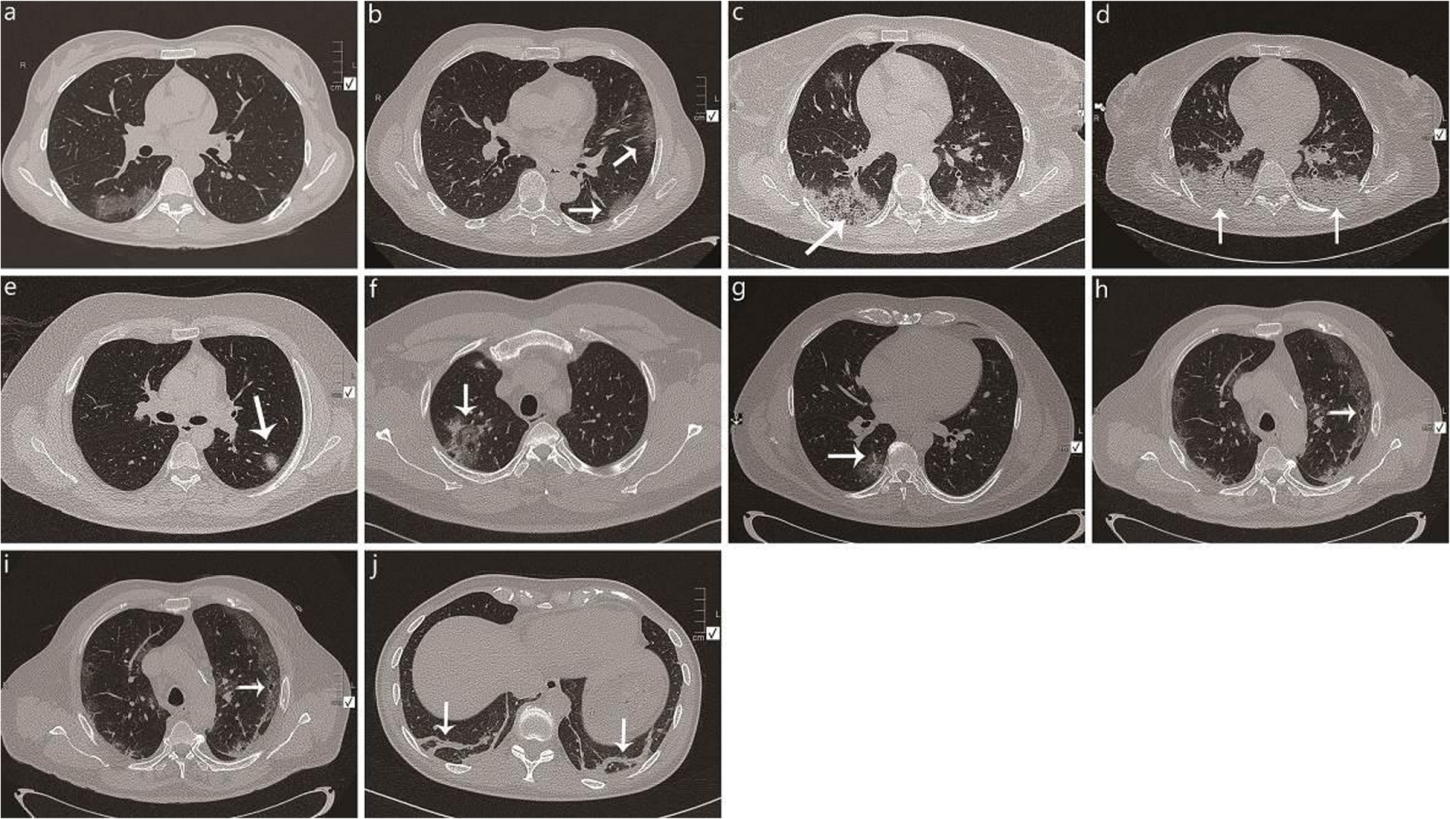
Typically radiological signs described for COVID-19. **a**. Ground-glass opacities distributed peripherally and subpleurally at right lower lobe. **b**. Bilaterally upper and lower lobe involvement. **c-d**. Crazy-paving pattern evolving into consolidation in day 4. **e**. Peripheral halo sign. **f**. Reverse halo sign. **g**. Vascular enlargement in the lesion (arrow). **h**. Vacuolar sign in the crazy-paving pattern (arrow). **i**. Prominence of interlobular septal thickening in the healing process. **j**. Thick fibrotic bands formed by the regression of infiltration areas in bilateral lower lobes (white arrows)

In a recent study, the positive CT finding rate was reported as high as 97% in COVID-19 cases confirmed by RT-PCR [11]. When these findings are encountered on CT, they support the diagnosis or raise the differential diagnosis in suspicious cases. Bilateral multilobar, lower-lobe dominated posterobasal and peripheral distribution, either patchy or round ground-glass opacities are the most common and pathognomonic findings of the disease [16–19]. Other findings include ground-glass opacities with surrounding consolidation termed as peripheral halo sign, interlobular septal thickening, crazy-paving pattern, ground-glass opacities accompanied by interlobular septal thickening with visualized background lung parenchyma, consolidation which distributed similarly as ground-glass opacity, and subpleural lines (Fig. 2) [4, 18]. Failure to protect the subpleural areas and pleural thickening at the point of contact is expected radiological features of the disease. In a meta-analysis that included 13 studies on CT findings of COVID-19, the most common of distributions were bilateral lung involvement (78.2%) and peripheral distribution (76.95%). The right lower lobe (87.21%), left lower lobe (81.41%), and bilateral lower lobes (65.22%) were the most affected lobes [20].

Generally, lesions in ground-glass opacity show bilateral-multilobar involvement in a peripheral-basal and subpleural distribution. The spread of lesions to the upper lobes may occur in a short time. Other forms of involvement are ground-glass opacities surrounded by fibrotic halo (reverse halo), vascular enlargement within the lesion, bronchiectasis or deformation or vascular dilatation (vascular enlargement sign) in the affected area, air bubble that usually develops within the lesion during the healing period (vacuolar sign) are typical imaging features for COVID-19 pneumonia (Fig. 2, Additional file 1) [21].

**Fig. 3:**
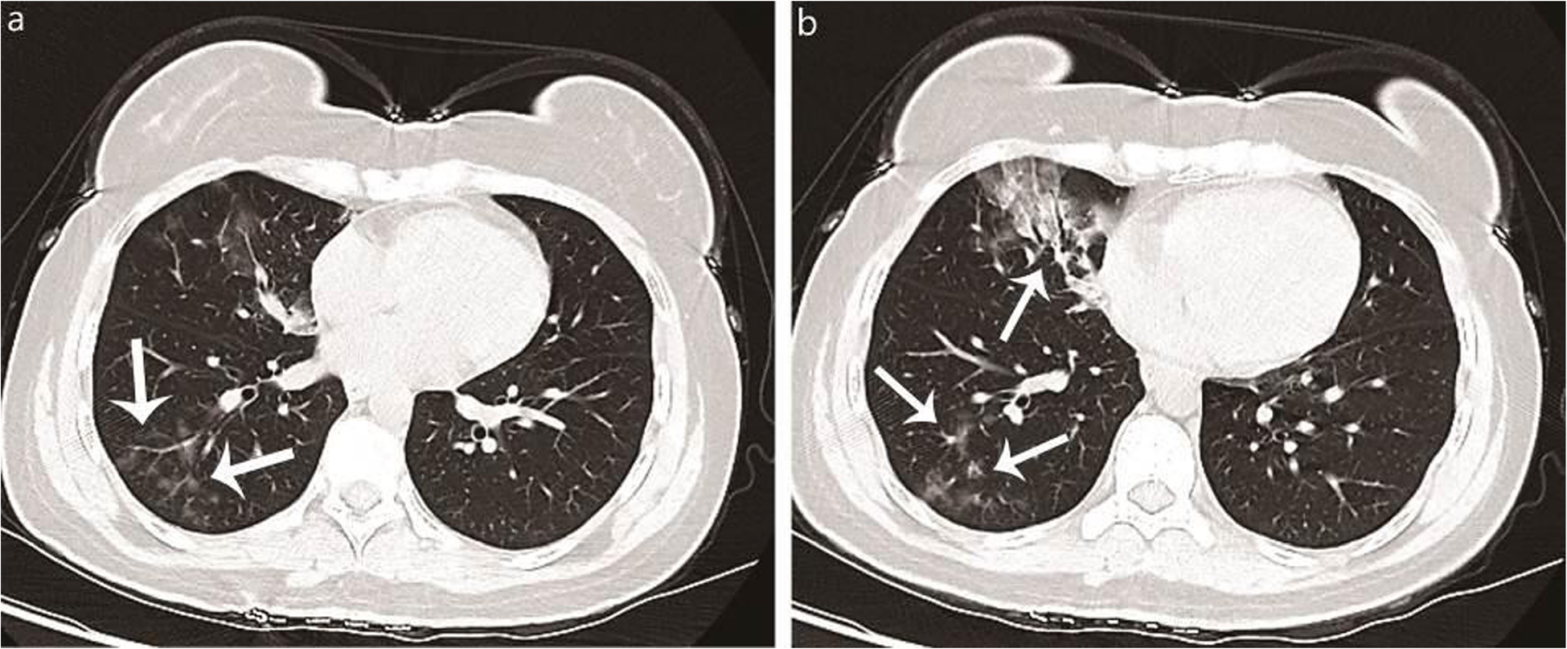
Atypical parenchymal infiltration RT-PCR positive case. **a**. Thirty-two years old, female case, RT-PCR(+), the atypical pattern for COVID-19, peribronchial distribution pattern and **b**. air bronchograms are in the consolidations accompanied by newly developing ground-glass opacities in the right upper lobe. Although this is not typical for the COVID-19 involvement pattern, atypical involvement is seen in this case with RT-PCR positive case

Non-COVID-19 related pneumonia is mostly in the form of consolidation that affects a single lobe accompanied by mediastinal lymphadenopathy and air bronchograms. The tree-in-bud view is generally detected in the early period or as an accompanying finding of ground-glass opacities (Fig. 3) [22].

### Stages of the disease and associated CT findings

#### Ultra early stage (incubation period, 1–2 weeks after contact)

Asymptomatic period. No imaging changes may be observed since the disease has not developed yet. Single/multiple focal ground-glass opacities, patchy consolidative densities, pulmonary nodules with ground-glass halos, air bronchograms.

#### Early stage (1–3 days after the onset of symptoms)

Symptomatic period. Single/multiple ground-glass opacities, ground-glass opacity ± interlobular septal thickening.

#### Rapid progression stage

Three to seven days after the onset of symptoms. Wide-mild consolidations and air bronchograms.

#### Consolidation stage

2nd week of symptoms onset. Regression can be seen in the size and density of the consolidations.

#### Dissipation stage

Two to three weeks later. Patchy consolidations, reticular opacities, bronchial wall thickening and interlobular septal thickening [23].

### Age-dependent chest CT findings

CT findings vary based on age [24–26]. The most common finding younger than 50 years of age is ground-glass opacity seen in 77% of cases. Consolidations are detected at a lower rate (23%). The most common finding in elderly cases is ground-glass opacities, too, with 55% of cases. The incidence of the disease in the form of consolidation has been reported in 45% of cases older than 50 years of age [17, 27]. In addition, atypical imaging findings for COVID-19 are more common in elderly patients (Additional file 1). This also underlines why the disease beginning with consolidation or atypical findings has a poor prognostic factor [17].

In 20% of pediatric cases, there is no imaging finding to suggest pneumonia [28]. In general, an infiltration pattern detected as ground-glass opacity is seen, similar to that of adults. On the contrary, it may start with direct consolidation and peripheral halo and progress rapidly in the form of atypical CT findings [29].

### Time dependent chest CT findings

#### Early stage (after the onset of symptoms (days 0–4)

The most common and initial finding is generally focal or patchy ground-glass opacities showing lower lobe peripheral-basal dominance. Although it is usually multiple, it may also appear as a single focus in the early period of the disease. Consolidations may accompany or appear later (42%). Consolidations can be seen alone or accompanied by peripheral ground-glass density (halo sign). Consolidations also show posterobasal, peripheral and lower lobe dominance similar to ground-glass opacities. 17% of the cases had no early CT findings.

#### Intermediate stage (days 5–13)

This is the stage where consolidations with bilateral-multilobar involvement, developing from a new focus, formed by conversion of the ground-glass opacity or increased in size are observed. Additionally, this is the phase where interlobular septal thickening is observed in ground-glass opacities, and the paving stone appearance (crazy-paving pattern) can be detected.

#### Late stage (> 14 days)

**Fig. 4:**
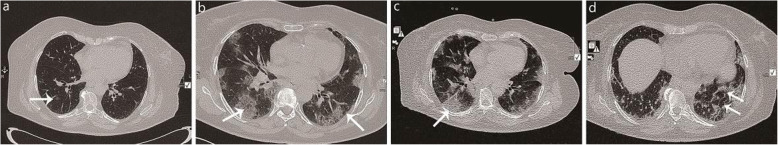
Serial images showing the expected evolution of the CT findings over time. **a**. CT exam dated March 17, 2020 shows viral parenchymal infiltration starting as a very low-density millimeter-sized ground-glass density in the central right lower lobe. **b**. CT exam dated March 23, 2020 shows interval progression into widespread ground-glass densities and crazy-paving pattern with peripheral and subpleural domination in both lower and upper lobes of the lung. **c**. CT exam dated March 26, 2020 shows interval evolution of the ground-glass and crazy-paving areas into consolidation, while some regressed directly without sequelae. **d**. CT exam dated April 2, 2020 during clinical recovery phase shows lesions regressed in the form of fibrotic bands and subpleural streaks

Parenchyma findings begin to regress, disappear completely or remain in the form of fibrotic bands. Although most of these fibrotic bands disappear completely, they have been reported to persist for a long time at a low rate (Fig. 4) [30].

**Fig. 5:**
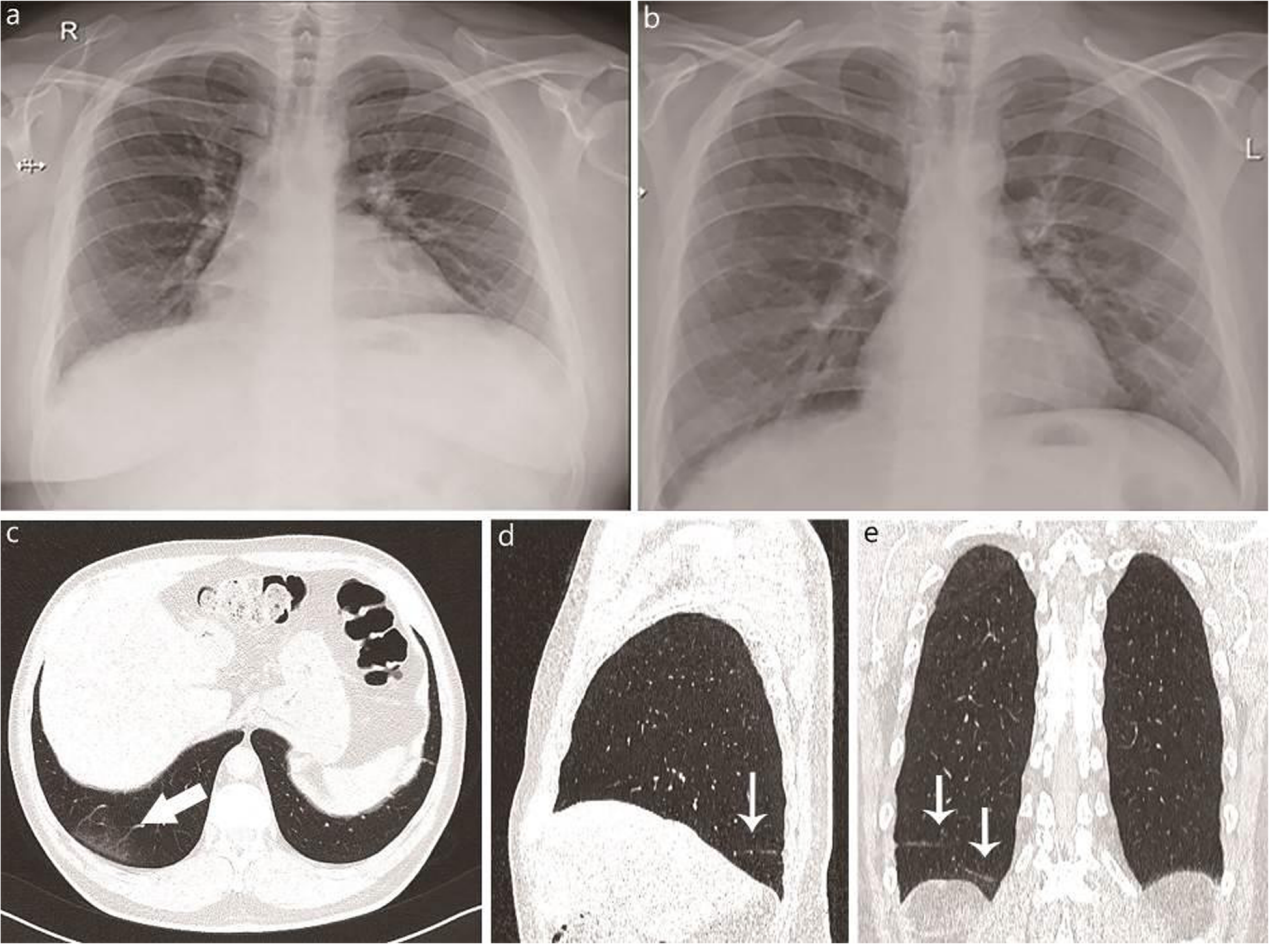
A 33-year-old male patient with band atelectasis mimicking GGO. Chest radiographs dated **a**. March 14, 2020 and **b**. March 22, 2020 are normal. **c-e**. CT images dated March 22, 2020 show focal patchy ground-glass density in the lower lobe of the right (thick arrow). Band atelectasis in axial sections, parenchymal distortions due to fibrotic disturbances may mimic the COVID-19 pattern (thin arrows). GGO. Ground glass opacity

**Fig. 6:**
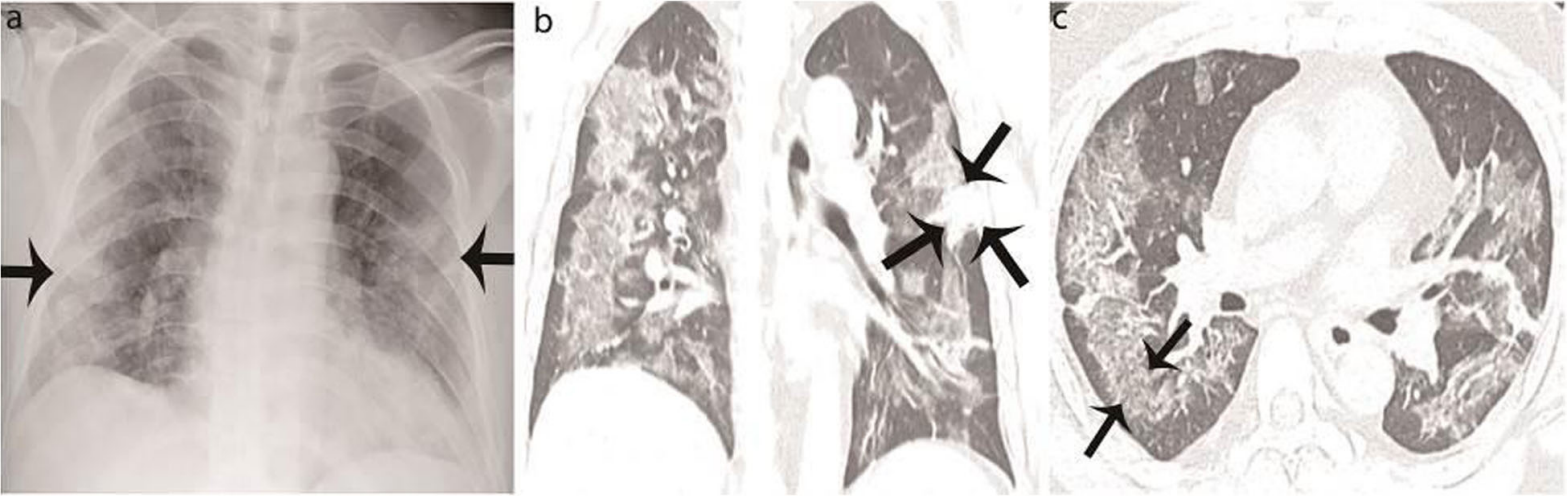
Visualization of the organized pneumonia pattern seen in the disease with X-ray and CT. **a**. A 52-year-old man. The chest X-ray obtained due to weakness shows peripheral-weighted focal density increases in the bilateral middle and lower zones (arrow). There is an organized pneumonia pattern in the form of crazy-paving accompanied by focal consolidation (arrow), suggesting diffuse alveolar damage and pneumonic infiltration in the coronal **b** and axial **c** CT sections of the same patient

While evaluating chest CT images, coronal and sagittal sections should be examined as well as axial sections. Multiplanar evaluation minimizes overlooking or overdiagnosing lesions that would otherwise be misinterpreted on a single section. Basal-peripheral-central distribution of the lesions and lower-upper lobe involvement can be evaluated better by multiplanar imaging (Figs. 5 and 6) [19, 31–33].

### Radiological prognostic factors on CT

Advanced age and comorbid disease are the most important prognostic factors for COVID-19 pneumonia [34, 35]. In addition, some poor prognostic factors have been identified based on CT imaging features [35]. The appearance of atypical findings, such as widespread consolidated areas, rapid infiltration into the upper lobes, pleural effusion, and mediastinal lymphadenopathy, are poor prognostic indicators. Rare findings, such as diffuse lesions, structural distortion, traction bronchiectasis, intrathoracic enlarged lymph nodes, and pleural effusion, are more common in the critical group [28, 35–37]. Multiple lobe involvement and subsegmental consolidations were detected to be the most common findings in patients who need subsequent intensive care units [30]. CT is important for disease progression as well as diagnosis. More serious findings detected by CT may be decisive in the follow-up and treatment algorithm (follow-up, isolation, drug administration, hospitalization) [16, 27, 38, 39].

### Relationship of CT findings with RT-PCR

**Fig. 7:**
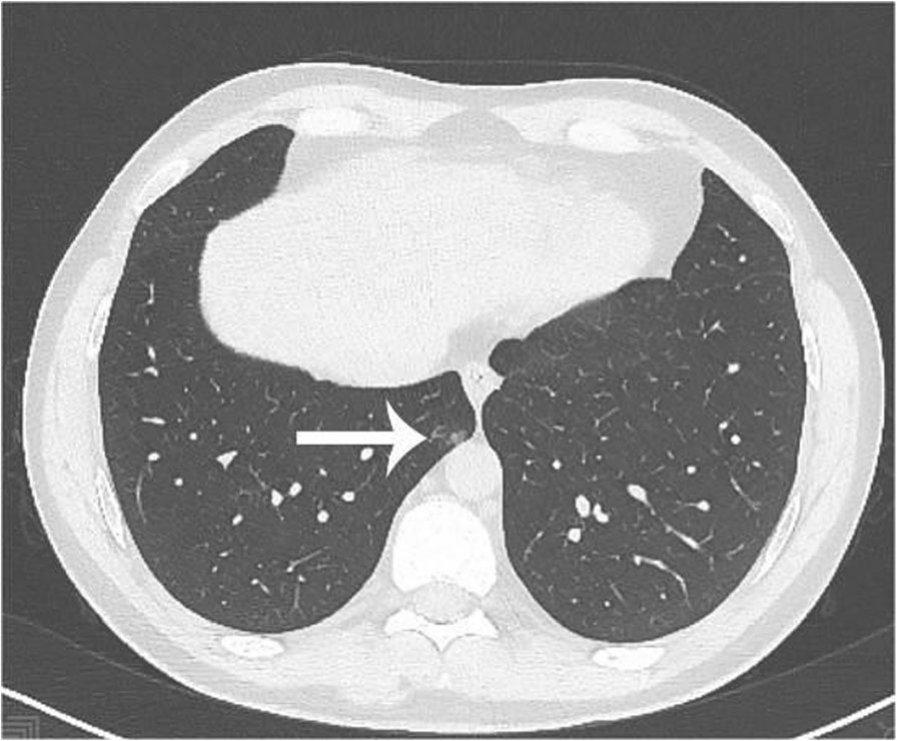
Positive CT finding in RT-PCR negative case with a history of contact. In this 34-year-old male patient with negative RT-PCR results and with COVID-19 positive family members. There is only a single millimeter-sized lesion in ground-glass opacity, subpleurally located in the right lobe medial lower lobe, significant in terms of involvement on CT (arrow)

Although RT-PCR sensitivity is reported as 70% on average, it varies according to the sampling method, the time of sampling, the provider collecting the specimen and the sensitivity of the kits [11, 40]. RT-PCR positivity is generally correlated with CT findings, with some exceptions. It was observed that RT-PCR became positive afterwards in patients with initial negative RT-PCR and positive CT findings [11, 41]. However, in a study of 167 patients, the CTs of seven patients who were RT-PCR positive were reported as normal, and one of these patients had positive CT findings developing 5 days later. In other words, both CT and RT-PCR, especially RT-PCR, can be negative in the early stages of the disease [42]. RT-PCR can be negative for up to 2–3 weeks from the onset of symptoms [11]. On the contrary, the CT findings become positive between 6 and 11 days (median 10th day) [11]. Patients without symptoms but with abnormal CT findings may develop symptoms 2–6 days later [4, 11]. If CT findings are present, there are management algorithms that accept cases as COVID-19 positive even if RT-PCR is negative. The most commonly accepted approach for the use of CT and RT-PCR during initial diagnosis is as follows: In the absence of typical-significant or suspicious findings for pneumonia, CT findings do not indicate COVID-19 positivity. It should be remembered that even if parenchymal involvement occurs within the first 3 days after symptoms begin, it is too early to reflect on imaging and imaging findings may appear after the 4th day. On the other hand, although it varies according to the local test type and sampling methods, the diagnostic value of CT taken, especially on the 3rd or later days after the onset of symptoms, is higher than RT-PCR (Fig. 7) [11, 41]. If there are clinical and laboratory findings or CT findings in RT-PCR negative case, the test should be repeated after 24 h [4, 11, 23]. In COVID-19 diagnosis, the possibility of misdiagnosis with CT was reported as 3.9%. In addition, COVID-19 may not be distinguished from pneumonia-related to other viral agents, especially SARS, Middle East respiratory syndrome (MERS) and adenovirus, based on CT findings [37]. However, in the presence of typical clinical findings, there is the potential to ensure correct triage in most of the cases when used with the appropriate indication [4, 42]. These cases should be considered positive for COVID-19, even if RT-PCR is negative [43].

### CT indications for COVID-19

**Fig. 8:**
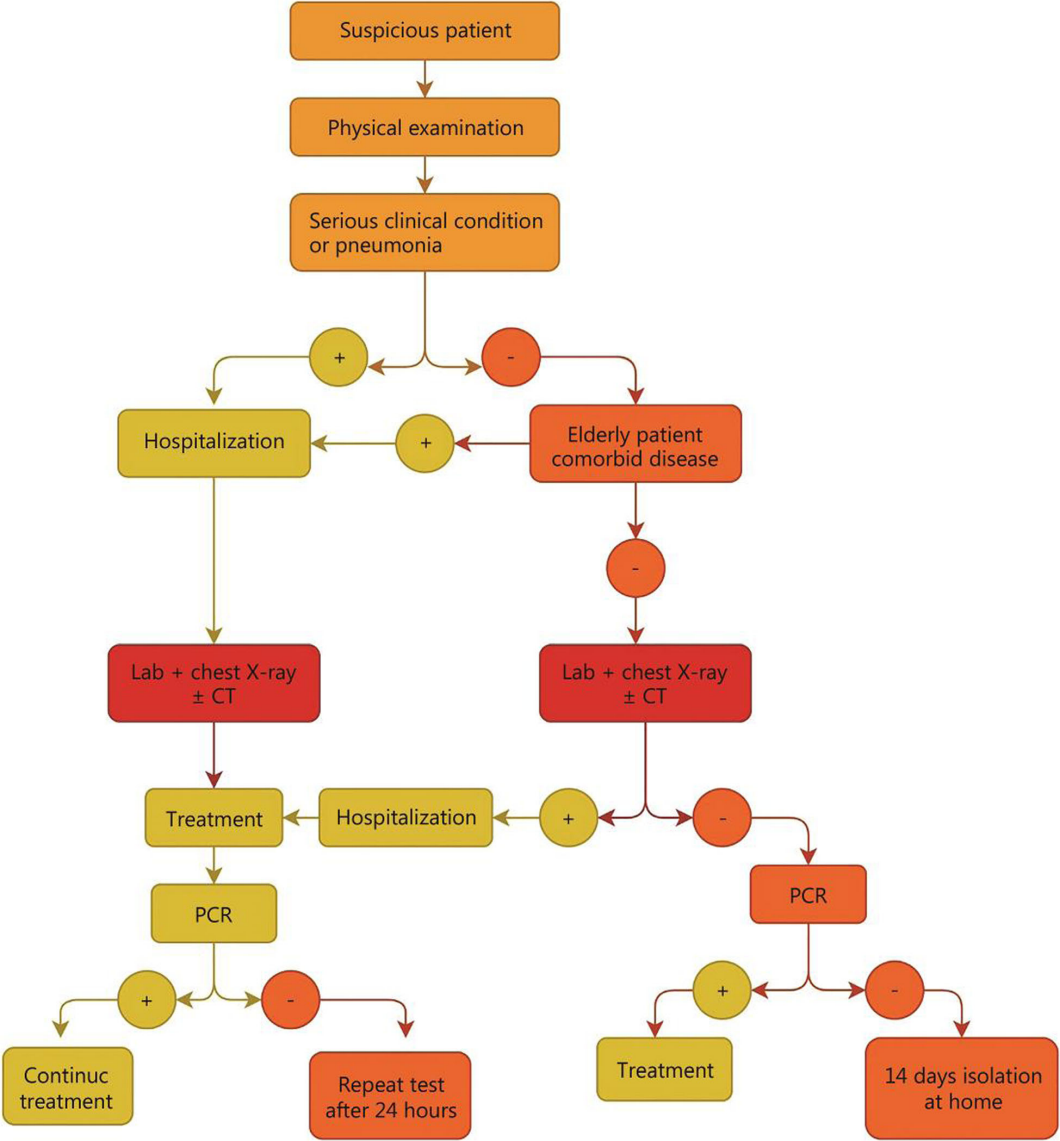
A simplified triage algorithm for COVID-19

Thorax CT is a sensitive diagnostic approach in the early period in RT-PCR test negative COVID-19 cases. When the RT-PCR test is not available, resources are scarce, or the COVID-19 test is negative, imaging is recommended to support the patient’s faster triage (Fig. 8) [43]. CT imaging is not recommended for COVID-19 positive with mild symptoms and without risk factors for disease progression [44]. CT imaging is recommended to assess secondary abnormalities, such as COVID-19 progression, pulmonary embolism, or secondary bacterial pneumonia, when the patient’s clinical condition worsens [4, 43–47].

Routine use of CT is not recommended for evaluation of response to treatment during follow-up of COVID-19 patients. Clinical and laboratory parameters are more sensitive in patient assessment. Radiological findings may not always correlate with the clinical status of patients. Nonetheless, radiological findings provide important insight into disease progression and development. If additional pathology that may affect the treatment decision is considered, imaging can be performed [4, 11, 43].

### Personal protection during and after CT screening

During and after CT screening of patients diagnosed with COVID-19, infection control protocol must be followed. In addition, every patient coming for CT should be considered as infected and personal protective equipment should be used by the technician and the patient. After the screening, the table should be disinfected; the CT room should be ventilated; the interval between patients should be at least 10–15 min. Equipment, such as overalls, gloves, masks and glasses, to be used by patients, as well as technicians, nurses and assistant personnel working in the tomography unit should be defined in detail by the institution. The rotation schedule of the technicians should be determined by working hours or the number of CT scans taken and should be reported to hospital management, hospital infection control units and radiology departments [23, 48–50].

### CT reporting of COVID-19

Thorax CT scans continue to be used at increasing rates all over the world due to the current pandemic. According to the explanations and recommendations of the Radiological Society of North America Expert Consensus (RSNA) on COVID-19 on April 1, 2020, the term COVID-19 pneumonia should not be used in the report; other viral pneumonia, especially influenza, drug intoxication, connective tissue diseases, hypersensitivity pneumonia, other causes that cause diffuse alveolar damage, idiopathic organized pneumonia may also create the same pattern as COVID-19. CT should be preferred as a supportive modality in the overall context of the clinical examination, laboratory findings, and PCR. In this way, it will be possible to minimize unnecessary anxiety of patients and their relatives while the diagnostic load of radiology is reduced [51].

RSNA Statement on Reporting Chest CT Findings related to COVID-19 and the British Society of Thoracic Imaging (BSTI) provided more discrete reporting samples by classifying the lesions better [51, 52]. We presented a sample report format in the Additional file 2. In the technical part, it is worth noting that the screenings were taken with a low dose and no contrast was administered. In the findings part, parenchymal, bronchial, pleural and the other changes should be described in detail. In the conclusion section, whether the involvement pattern suggests pandemic type viral infiltration should be stated. We use the RSNA recommendations in the results-suggestions sections of the report in our own clinic.

In the literature and on the web, there are many different classification and reporting formats. Each institute can edit and tailor one of these formats based on the institution’s needs [51–53].

## Conclusion

Thoracic radiological imaging has a critical role in the diagnosis and management of COVID-19 pneumonia. The first goal in chest imaging should be “to detect COVID-19 pneumonia” and “differentiate cases without lung involvement”. Another preferential goal should be to identify specific patterns that have the potential to predict the disease course. This will be extremely useful, especially for cases with advanced age and comorbidities. Low-dose CT can be used to reveal normal, typical, atypical parenchymal findings and to evaluate patients’ follow-up and treatment response, especially in appropriate indications. In particular, clear identification of the findings part and standardization of the result-recommendation part of the CT reports will provide fast and effective communication between radiologists and clinicians.

## Supplementary Information

**Additional file 1:.** CT imaging findings for COVID-19.

**Additional file 2:.** An institutional sample organized template of the report format.

## Data Availability

Not applicable.

## Abbreviations

2019-nCoV: Novel coronavirus
BSTI: British Society of Thoracic Imaging
COVID-19: Coronavirus disease 2019
CT: Computed tomography
RSNA: Radiological Society of North America Expert Consensus
SARS-CoV-2: Severe acute respiratory syndrome coronavirus 2
X-ray: Direct radiography
