# Supplementary material for: A practical approach to imaging characteristics and standardized reporting of COVID-19: a radiologic review
Source: Mil Med Res. 2021 Jan 24;8:7. doi: 10.1186/s40779-021-00301-y (PMC7826494; doi:10.1186/s40779-021-00301-y)
Supplement: Supplementary file 1 — Additional file 1:. CT imaging findings for COVID-19. [file 40779_2021_301_MOESM1_ESM.docx]

**Additional file 1** CT imaging findings for COVID-19

| **Significant CT findings for COVID-19 than other viral agents** |
| --- |
| Bilateral, multilobar, especially lower lobes |
| Progression of lesions in number and size and rise to upper lobes or even apex |
| Subpleural-peripheral parenchymal infiltration |
| Ground-glass opacity-consolidation combinations (mostly peripheral and subpleural) |
| Peripheral halo finding in ground-glass opacity around the consolidations |
| Peripheral more intense consolidation or area with central ground-glass opacity surrounded by fibrotic halo (reverse halo sign) |
| Ground-glass opacities accompanied by interlobular septal thickening (Crazy-paving pattern) |
| Vascular enlargement sign |
| Bronchial deformation sign, tractional bronchiectasis |
| Discrete pulmonary nodules (more common in the upper-middle zone and bronchovascular orientation) |
| **Non-typical CT imaging findings for COVID-19** |
| Pleural effusion |
| Pericardial effusion |
| Lymphadenopathy |
| Cavitation (not to be confused with the bubble sign) |
| Pneumothorax |
| Distribution of infiltrations in the unilateral/ peribroncovascular/ centrilobular/ bronchopneumonia pattern, tree-in-bud |
| Consolidation(s) only |
| Lesions only in the upper lobes |
