# Supplementary material for: A practical approach to imaging characteristics and standardized reporting of COVID-19: a radiologic review
Source: Mil Med Res. 2021 Jan 24;8:7. doi: 10.1186/s40779-021-00301-y (PMC7826494; doi:10.1186/s40779-021-00301-y)
Supplement: Supplementary file 2 — Additional file 2:. An institutional sample organized template of the report format. [file 40779_2021_301_MOESM2_ESM.docx]

**Additional file 2** An institutional sample organized template of the report format

**Examination of non-contrast low-dose chest CT**

**Technique**: This imaging consisted of low-dose non-contrast CT sections obtained due to a viral pandemic that could produce a specific pulmonary pattern. The reporting format was shaped in accordance with these pandemic conditions. Pandemic type viral pneumonic infiltration was considered to be significant for lung involvement, together with special patterns. It was considered to be significant for pulmonary involvement with specific patterns indicating pandemic type viral pneumonic infiltration.

**Findings**:

(of RSNA or BSTI classification recommendation)
